# Interaction between Bottlebrush Polymers and Phospholipid Membranes in Solutions

**DOI:** 10.3390/polym12123033

**Published:** 2020-12-17

**Authors:** Xiaoyong Dai, Yongyun Ji, Zhenguo Wang, Linli He, Xianghong Wang, Shiben Li

**Affiliations:** Department of Physics, Wenzhou University, Wenzhou 325035, China; 15651725183@163.com (X.D.); yyji@wzu.edu.cn (Y.J.); linlihe@wzu.edu.cn (L.H.); wxh@wzvtc.cn (X.W.)

**Keywords:** phospholipid membrane, bottlebrush, force

## Abstract

In this work, the interactions between bottlebrush polymers and phospholipid membranes were investigated using dissipative particle dynamics simulations. The weak and strong adsorption phenomena between the polymers and membranes were examined by calculating the system parameters. A spring model was introduced to explain the variances in the shape factors and the radius of gyration of the bottlebrush polymers, as well as the order parameters of the phospholipid membrane in the pulling processes. This work provides further understanding for the application of bottlebrush polymers in biological processes.

## 1. Introduction

Cell membranes comprise three major types of lipids, namely, phospholipids, cholesterol, and glycolipids, in which the phospholipids are one of the most abundant types [1]. The phospholipid membrane separates the internal and external environments of the cell and controls the exchange of substances inside and outside the cell because of its semipermeability. This property plays an important role in the protection of cells and organelles. Therefore, the interactions between the polymers and lipid membranes and those between the lipid membranes within the metal nanoparticles are unavoidable in biological processes [2]. Notably, research in the interactions between the phospholipid membrane and the polymers has attracted extensive attention, thus promoting the further development of medical carriers [3,4,5].

For example, Houang et al. used the all-atom model to investigate the interactions between the amphiphilic triblock copolymers between the phospholipid membranes, where the polymers are composed of poly(propylene oxide) as the core and two linear poly(ethylene oxide) chains on both sides [6]. While the insertion of triblock copolymers into the lipid bilayer increases the absolute apparent lateral pressure required for bilayer rupture, it also decreases the lateral pressure necessary for membrane rupture [6]. Moreover, Raczyński et al. investigated the interactions of carbon-based materials with phospholipid membranes by inserting graphene (GN) sheets of different sizes into a bilayer [7,8,9,10,11]. The phospholipid membrane undergoes an effective self-sealing process while the membrane is severely damaged, indicating that the GN sheet is a potential medical drug carrier [11].

For the other polymers such as bottlebrush polymers, the interaction may be different from those observed in triblock copolymers or carbon-based materials. Bottlebrush polymers are a special kind of polymers with unique structures, which consist of a linear backbone and polymeric side chains. Bottlebrush molecules will have many potential applications due to their special chain topology, which is different from traditional linear polymers [12], including molecular pressure sensors [13], pH-sensitive molecular probes [14], super-soft elastomers [15], antifouling, and stimuli-responsive surface coatings and lubricants [16,17,18,19,20,21]. Notably, the role of the bottlebrush molecules in the medical field has aroused the interest in the interaction between bottlebrush polymers and phospholipid membranes. Garle et al. investigated the interactions between vesicle membrane and the bottlebrush polymers, whose side chains consisted of polyethylene glycol (PEG); they found that compared with ungrafted cationic polymers, molecular brushes with PEG as the side chain have significant film penetration ability because the side chains are more likely to be inserted into the phospholipid membranes [22]. Golder et al. obtained a similar conclusion that the increased density of PEG side chains helped the nanoparticles enter the cell [23]. Liu et al. used the electrostatic interaction between the polyelectrolyte brush and the oppositely charged membrane vesicles to form a flat cell membrane double layer from the vesicles [24]. These results indicated that the brush polymers will effectively interact with the membranes. Moreover, studies on other bottlebrush-like structures have also obtained similar results [25,26]. In addition, Wang et al. investigated the self-assembled microstructures of bottlebrush polymers and the interactions with lipid membrane in aqueous solution; their findings indicated that bottlebrush polymers are efficient medical carriers in solutions [27].

The interaction between the bottlebrush polymers and phospholipid membranes sometimes results in novel properties in the interaction mechanism. This fact requires a further understanding of the influence of bottlebrush polymers interacting on the phospholipid membranes. In the current research, we focus on the interaction between a single bottlebrush polymer chain and the phospholipid membrane. In addition, the bottlebrush polymers are adsorbed into the membrane surfaces, and the weak and strong adsorption phenomena are investigated in the pulling processes. The pulling forces are varied to calculate the relevant system parameters, such as the shape factors and radius of gyration, and the order parameters are also used to observe the interaction processes. In the next section, we introduce the model and the methods used. In the third section, we discuss and analyze the simulation results, and then summarize the corresponding conclusions.

## 2. Model and Method

### 2.1. Methodology

In the current work, we used the dissipative-particle dynamic (DPD) method, which has been widely used in soft material and biomembrane systems, to simulate the polymer and membrane systems [27,28,29,30,31]. In a DPD simulation in [32], a small group of atoms was treated as a single bead located at the center of mass of the group. Through this method, the amount of calculation was greatly reduced while maintaining accuracy. Here, we briefly describe the basic formulations in the DPD method. In the system, we marked a pair of random beads as *i* and *j*. Three types of forces exist in the pair of *i*-th and *j*-th beads. The conservation force, $F_{ij}^{C}$, represents the excluded volume effect, while the dissipative force, $F_{ij}^{D}$, represents the viscous drag between moving beads, and the random force, $F_{ij}^{R}$, represents the stochastic impulse. Therefore, the total forces acting on *i*-th bead can be expressed as follows:

(1) $$F_{i}=\underset{i\neq j}{\sum }\left(F_{ij}^{C}+F_{ij}^{D}+F_{ij}^{R}\right)=\underset{i\neq j}{\sum }\left[a_{ij}w\left(r_{ij}\right)r_{0ij}-\gamma w^{2}\left(r_{ij}\right)\left(r_{0ij}\cdot v_{ij}\right)r_{0ij}+\sigma w\left(r_{ij}\right)\zeta_{ij}\Delta t^{-1/2}r_{0ij}\right]$$

where $a_{ij}$ is the maximum repulsive force, while $r_{ij}$ and $v_{ij}$ denote the distance and the relative velocity between the *i*-th and *j*-th beads, respectively, with the unit vector $r_{0ij}$. In addition, $\zeta_{ij}$ denotes a random number chosen from a uniform random distribution and in an independent manner for each pair of particles with a zero mean and unit variance of one [33]. The parameters γ and σ are related by $\sigma^{2}=2\gamma k_{B}T$, where $k_{B}$ is the Boltzmann constant, where γ=4.5 and σ=3.0 in our simulation [28,32]. Lastly, $w\left(r_{ij}\right)$ is the normalized distribution function that can be expressed as follows:

(2) $$w\left(r_{ij}\right)=\left\{ \begin{array}{ll} 1-\frac{r_{ij}}{r_{c}} & r_{ij}<r_{c}\\ 0 & r_{ij}>r_{c} \end{array}\right.$$

where $r_{c}$ is the cut-off radius.

### 2.2. Model

**Figure 1:**
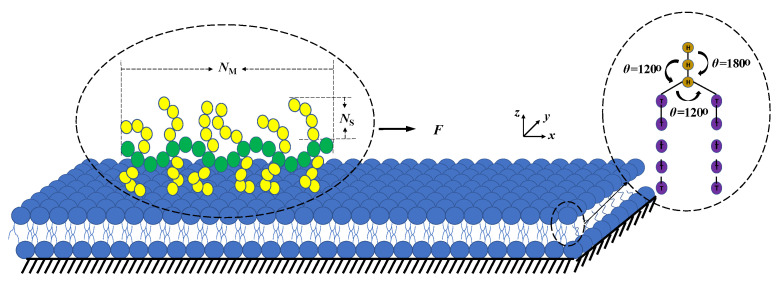
Schematic view of the phospholipid membrane and bottlebrush polymer model. The coarse-grained phospholipid polymer chain is constructed by one hydrophilic chain (the upper gold beads), and the two hydrophobic chains (the bottom purple tail beads). The bottlebrush polymer is placed in the phospholipid membrane where the main chain is marked as green and side chains are marked as yellow. The membrane is supported by a substrate where two heads beads are fixed in the lowermost layers. The pulling forces are applied along the *x*-direction.

Based on the coarse grain (CG) model, we showed a concise model diagram in Figure 1. The phospholipid membrane was constructed by two kinds of beads, and here we employed a universal model with two tail linear chains and one head chain [34]. The hydrophilic heads were represented by green beads, and hydrophobic tails were represented by purple beads. An additional elastic harmonic force was used to connect two consecutive beads:

(3) $$F_{ij}=k_{s}\left(1-\frac{r_{ij}}{r_{s}}\right)r_{0ij}$$

where $k_{s}$ and $r_{s}$ are the spring constant and equilibrium bond length, respectively. We used the parameters $k_{s}=120.0$ and $r_{s}=0.7r_{c}$, which are similar to those in the previous studies [27,35]. We applied another force on two consecutive bonds to ensure the bending resistance for the chains, and it is given as follows:

(4) $$F^{\theta }=-\nabla \left[k_{\theta }{\left(\theta -\theta_{0}\right)}^{2}\right]$$

here, $k_{\theta }$, θ, and $\theta_{0}$ are the bending constant, inclination angle, and equilibrium angle, respectively. In the current simulations, we used $k_{\theta }=6$ and θ=π for three consecutive head beads or tail beads and $\theta =\frac{2}{3}\pi$ for three consecutive beads at the connective point, as shown in Figure 1. In the membrane model, $a_{ij}=25$ was taken for the same types of beads, and $a_{ij}=100$ for the different types of beads, respectively, similar to previous works [27,28]. In our model, the membrane is supported by a substrate, which keeps the two head beads static in the lowermost layers of the phospholipid membrane. We have tried several cases with various numbers of fixed head beads, and the results indicated that the two fixed head beads in the lowermost layers are suitable in the current simulations.

For the bottlebrush polymers, we took $k_{s}=200.0$ and $r_{s}=0.5r_{c}$ to prevent the disintegration of bottlebrush molecules under the pulling force. We used $k_{\theta }=4.5$ and θ=π for three consecutive beads in the main chain or sidechains. In the bottlebrush polymer model, we used $a_{ij}=15$ for the same type of beads and $a_{ij}=50$ for the two beads of different types, where the green beads represent the mainchain while the yellow beads represent sidechains, as shown in Figure 1. We tried several sets of DPD parameters, and the current set parameters are suitable to observe the pulling effects. On the other hand, we are aware that the DPD parameters can be linked to the χ parameters in the Flory–Huggins type models [33,36]. Here, the DPD parameters used are similar to those used in the previous studies [27,28,35,37]. The mainchain length ($N_{M}$) was set to 100 beads, while the total number of sidechains ($n_{S}$) was 98, where one single sidechain consisted of 10 beads, i.e., $N_{S}=10$. Each sidechain was individually grafted to every particle on the main chain except the head and tail particles. In the beginning of pulling process, the bottlebrush polymer is randomly distributed on one end of membrane surface.

### 2.3. Simulation Parameters

In the DPD simulation, energy can be normalized by $k_{B}T$, and the length can be normalized by the cut-off radius $r_{c}$ Here, $r_{c}={\left(\rho V_{b}\right)}^{1/3}$, where $V_{b}$ is the volume of one bead, and ρ is the particle density. Considering that these values have been used in previous works, we set ρ=3, $V_{b}=0.03{\;\mathrm{nm}}^{3}$, and $r_{c}=0.5\;\mathrm{nm}$ [27,38]. In the current simulation, a modified version of the velocity–Verlet algorithm was incorporated to integrate the equations of motion with a time step of Δt=0.005τ, where τ is the natural unit of time defined as follows:

(5) $$\tau =r_{c}\sqrt{m/k_{B}T}$$

here, m is the mass of the bead [39]. All simulations were performed on an NVT ensemble by using LAMMPS [27,34,37]. To clearly observe the pulling process, we performed the simulation within a cuboid box with a volume of $400r_{c}\times 30r_{c}\times 20r_{c}$, whose three directions were all applied by the periodic boundary conditions. Two phenomena, namely weak and strong adsorptions, which are the interactions between the bottlebrush polymers and the head tail for the upper layers in the bilayer membranes of $a_{\mathrm{BM}}=-5$ and $a_{\mathrm{BM}}=-20$, were defined in the current simulation. Pulling forces F whose directions are along the *x*-axis are applied in each bead for the bottlebrush polymers in the range of 0.8–2.4. Specifically, each bead, either in the side chain or in the main chain, is applied by a force; more specifically, there are many forces applied to one polymer chain. This manner can probably be realized in the polyelectrolytes in the experiments where the orientation of the electric field is specified [40,41]. On the other hand, this manner of applying force can provide more obvious effects in the pulling process. Actually, because the simulation box is large enough in the *x*-direction, the periodic boundary condition disappears in this direction. The results were obtained from one single simulation since the length of the phospholipid membrane is large enough to ensure that the polymers make full effects with the membrane in a single pulling process.

## 3. Results and Discussion

**Figure 2:**
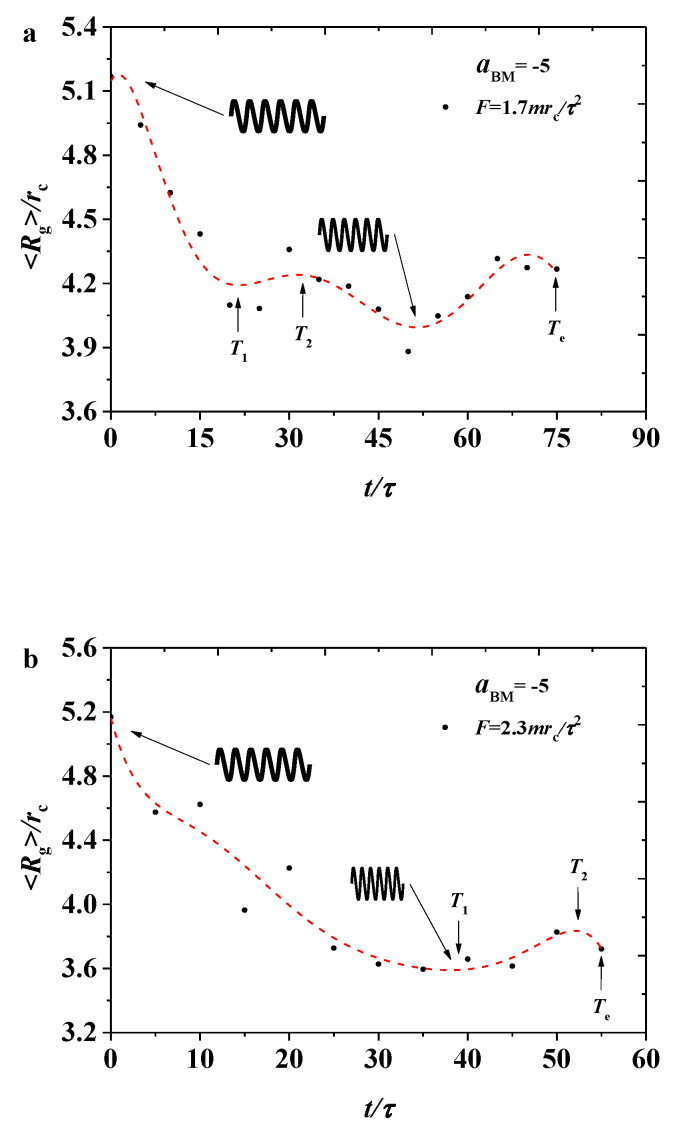
Variation of gyration radius for the bottlebrush polymers $\langle R_{g}\rangle$ in the weak adsorption cases of $a_{\mathrm{BM}}=-5$, where the red dotted lines are the fitting results with (**a**) the pulling force of $F=1.7mr_{c}/\tau^{2}$ and (**b**) the pulling force of $F=2.3mr_{c}/\tau^{2}$, respectively.

**Figure 3:**
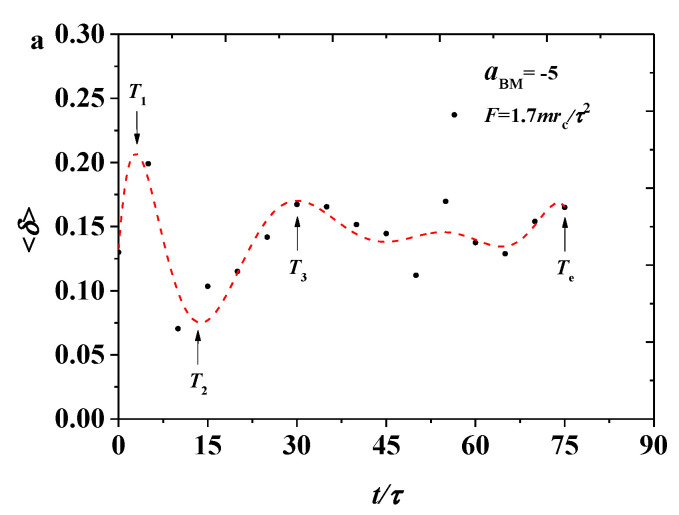

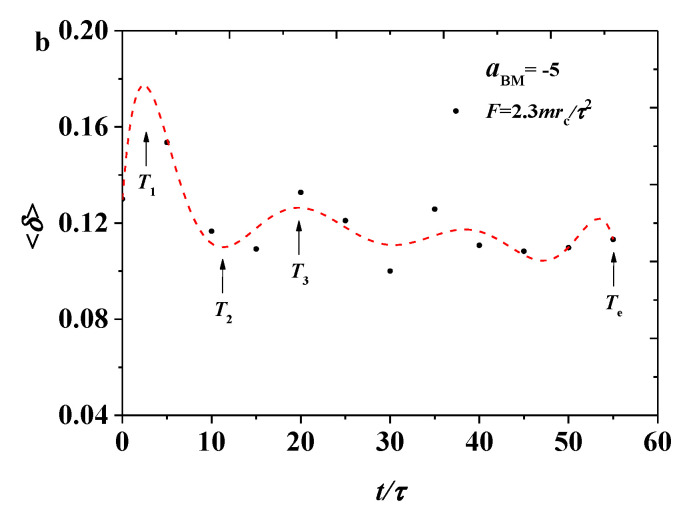
Variation of the shape factor 〈δ〉 for the bottlebrush polymers in the weak adsorption cases of $a_{\mathrm{BM}}=-5$, where the red dotted lines are the fitting results with (**a**) the pulling force of $F=1.7mr_{c}/\tau^{2}$ and (**b**) the pulling force of $F=2.3mr_{c}/\tau^{2}$, respectively.

**Figure 4:**
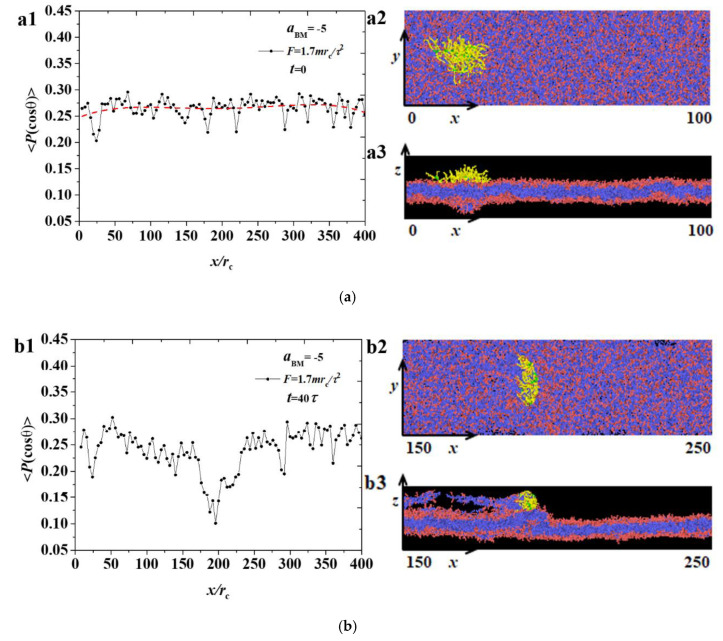

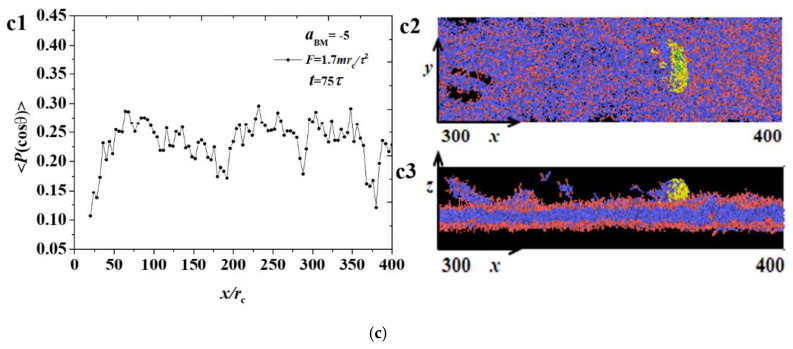
The distribution of order parameters 〈P(cosθ)〉 for the phospholipid membrane in the weak adsorption cases of $a_{\mathrm{BM}}=-5$ and $F=1.7mr_{c}/\tau^{2}$, where the membrane structures are also displayed. Three time stages, (**a**) t=0τ, (**b**) t=40τ, and (**c**) t=75τ, are demonstrated.

**Figure 5:**
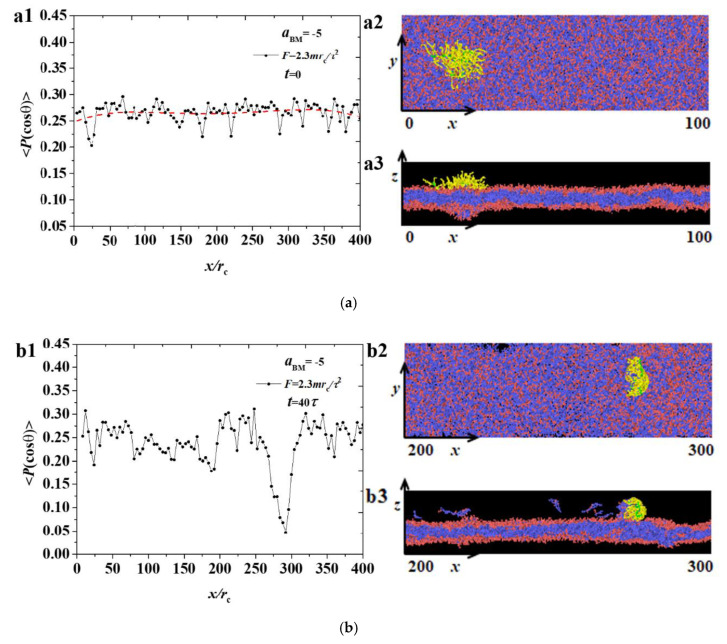

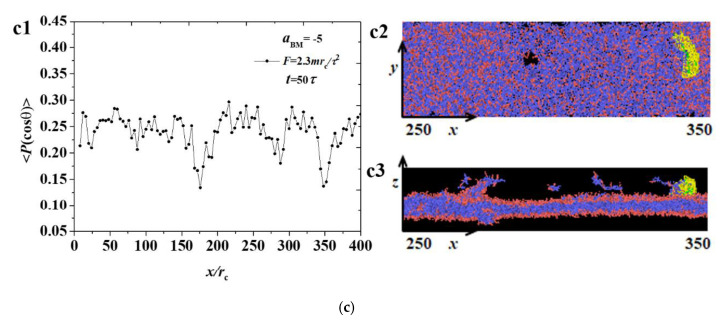
The distribution of order parameters 〈P(cosθ)〉 for the phospholipid membrane in the weak adsorption cases of $a_{\mathrm{BM}}=-5$ and $F=2.3mr_{c}/\tau^{2}$, where the membrane structures are also displayed. Three time stages, (**a**) t=0τ, (**b**) t=40τ, and (**c**) t=50τ, are demonstrated.

**Figure 6:**
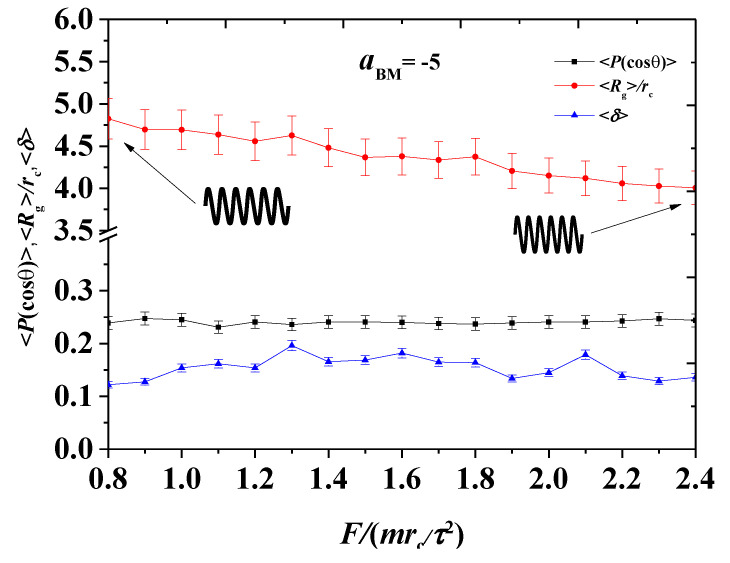
The system parameters as functions of the pulling forces in the weak adsorption cases of $a_{\mathrm{BM}}=-5$. The red dots and blue dots are the average radius of gyration $\langle R_{g}\rangle$ and average shape factor 〈δ〉 for the bottlebrush polymers, respectively, while the black dots denote the order parameters 〈P(cosθ)〉 for the phospholipid membrane.

**Figure 7:**
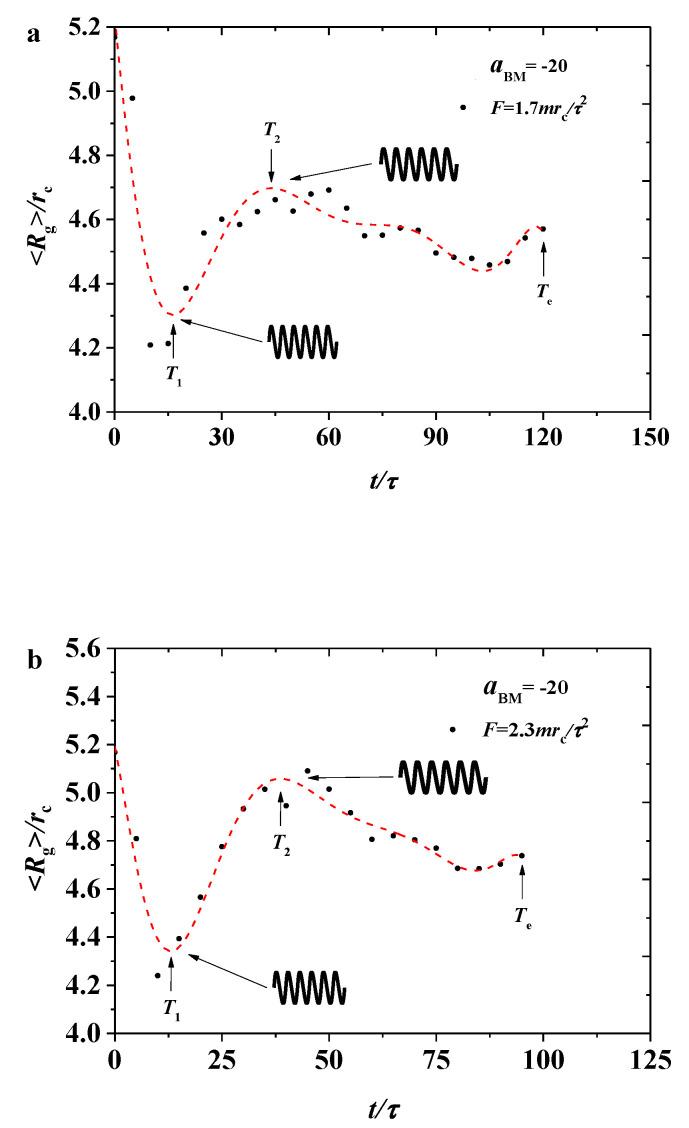
Variation of gyration radius for the bottlebrush polymers $\langle R_{g}\rangle$ in the strong adsorption cases of $a_{\mathrm{BM}}=-20$, where the red dotted lines indicate the fitting results with (**a**) the pulling force of $F=1.7mr_{c}/\tau^{2}$ and (**b**) the pulling force of $F=2.3mr_{c}/\tau^{2}$, respectively.

**Figure 8:**
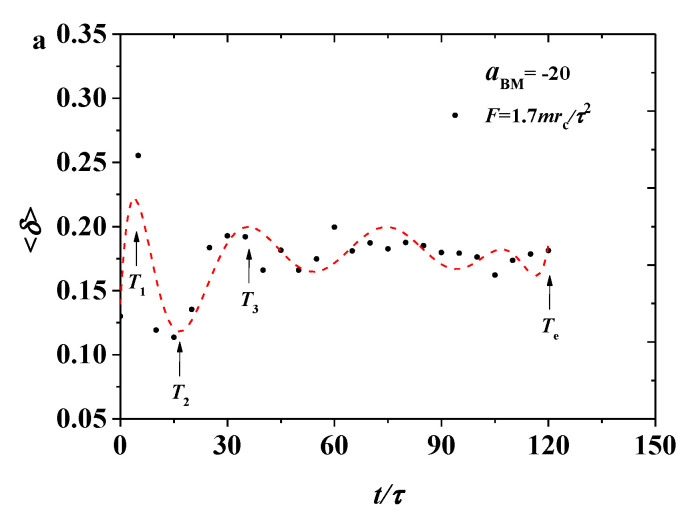

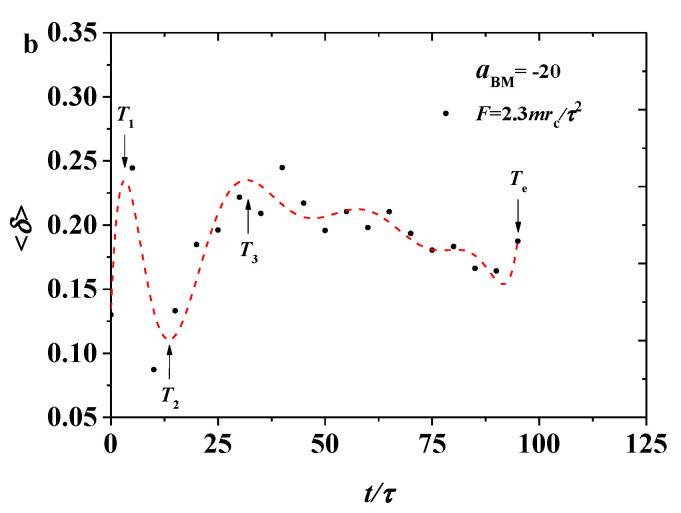
Variation of the shape factor 〈δ〉 for the bottlebrush polymers in the strong adsorption cases of $a_{\mathrm{BM}}=-20$, where the red dotted lines are the fitting results with (**a**) the pulling force of $F=1.7mr_{c}/\tau^{2}$ and (**b**) the pulling force of $F=2.3mr_{c}/\tau^{2}$, respectively.

**Figure 9:**
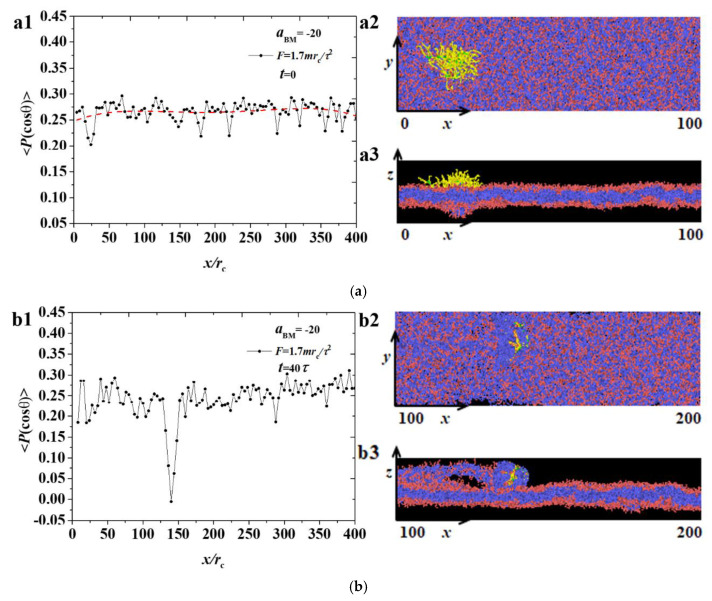

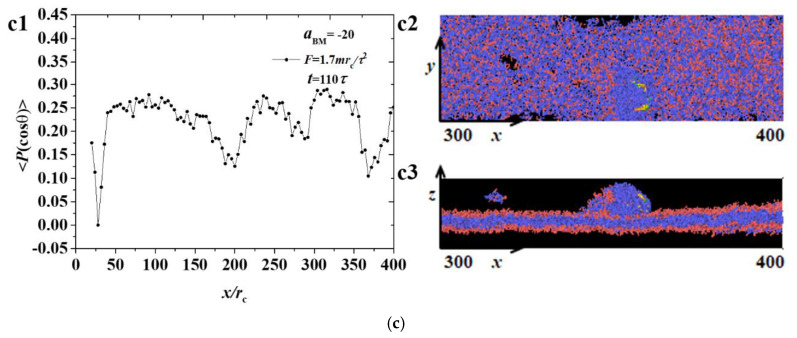
The distribution of order parameters 〈P(cosθ)〉 for the phospholipid membrane in the strong adsorption cases of $a_{\mathrm{BM}}=-20$ and $F=1.7mr_{c}/\tau^{2}$, where the membrane structures are also displayed. Three time stages, (**a**) t=0τ, (**b**) t=40τ, and (**c**) t=110τ, are demonstrated.

**Figure 10:**
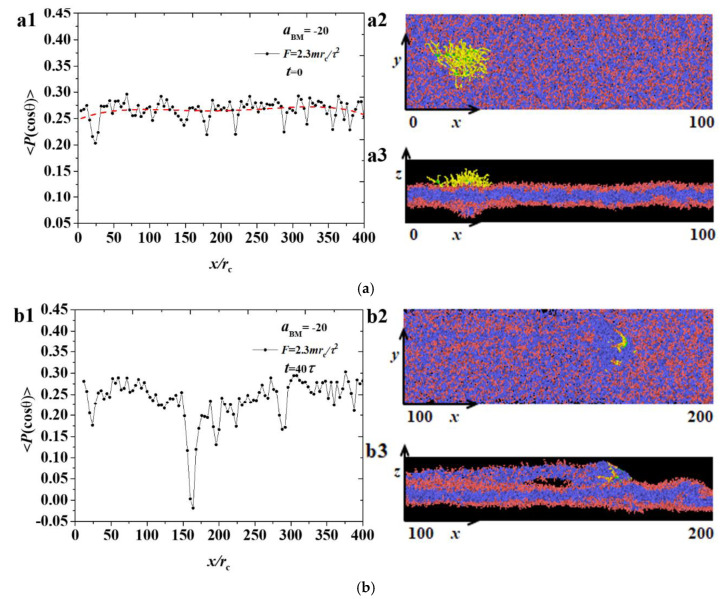

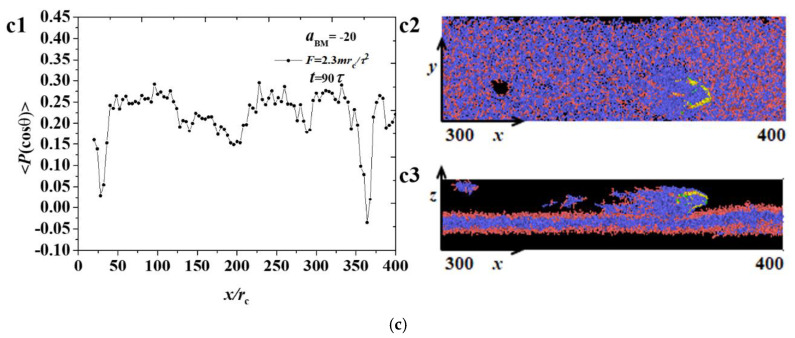
The distribution of order parameters 〈P(cosθ)〉 for the phospholipid membrane in the strong adsorption cases of $a_{\mathrm{BM}}=-20$ and $F=2.3mr_{c}/\tau^{2}$, where the membrane structures are also displayed. Three time stages, (**a**) t=0τ, (**b**) t=40τ, and (**c**) t=90τ, are demonstrated.

**Figure 11:**
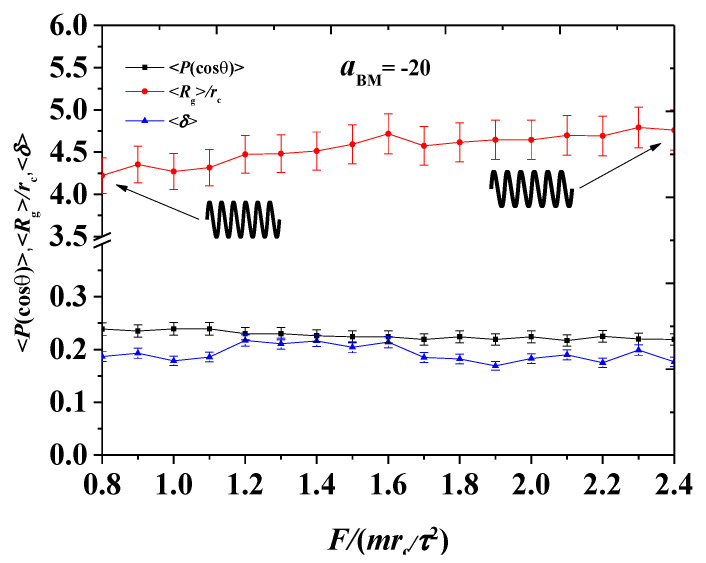
The system parameters as functions of the pulling forces in the strong adsorption cases of $a_{\mathrm{BM}}=-20$. The red dots and blue dots are the average radius of gyration $\langle R_{g}\rangle$ and average shape factor 〈δ〉 for the bottlebrush polymers, respectively, while the black dots denote the order parameters 〈P(cosθ)〉 for the phospholipid membrane.

The simulation results were sorted into two parts, namely, the weak and strong adsorption interactions between the bottlebrush polymers and the hydrophilic head of the phospholipid membrane. In each case, both pulling forces were applied in the bottlebrush polymers. In this section, we discuss the shape factors and radius of gyration for the bottlebrush polymers, the order parameters for the phospholipid membranes in the weak case ($a_{\mathrm{BM}}=-5$, Figure 2, Figure 3, Figure 4, Figure 5 and Figure 6), and those parameters in the strong case ($a_{\mathrm{BM}}=-20$, Figure 7, Figure 8, Figure 9, Figure 10 and Figure 11).

### 3.1. Weak Adsorption

#### 3.1.1. Gyration Radius

The gyration radius is an important parameter to understand the polymer size in the dynamic process [42]. The average radius of gyration $\langle R_{g}\rangle$ can be defined as follows:

(6) $$\langle R_{g}^{2}\rangle =\frac{1}{N}\sum_{i=1}^{N}\langle {\left(r_{i}-r_{c}\right)}^{2}\rangle$$

To study the influence of different pulling forces on $\langle R_{g}\rangle$, we selected two different pulling forces, $F=1.7mr_{c}/\tau^{2}$ and $F=2.3mr_{c}/\tau^{2}$, in the pulling processes, as shown in Figure 2, where the dotted lines are plotted for the variance trends. For convenience, we labelled several special times, such as $T_{1}$, $T_{2}$, and $T_{e}$ in Figure 2, where $T_{e}$ means the end time for the pulling processes stopped as the membranes is broken. For these two processes, the $\langle R_{g}\rangle$ of the bottlebrush polymers gradually decreased in a fluctuating manner. Under the pulling forces, the bottlebrush chains begin in the relaxed state with a relatively large $\langle R_{g}\rangle$, and then the unmatched movements between the chains caused the chain beads to be crowded, thus decreasing the chain size. The differences between these cases were obvious, as shown in Figure 2a,b. Firstly, the relatively small force leads to a quite complicated process with two local maximum peaks, while the larger force results in only one local maximum peak. Hence, the bottlebrush chain configuration undergoes a complicated variance under a certain value of force. By comparing the time of $T_{1}$, we can find that a small force leads the shorter time for the bottlebrush chains to reach the first local minimum value, and the value is larger than that in the larger force. In addition, the end time of $T_{e}$ in Figure 2a is longer. Secondly, these two processes can be explained by a spring model for the bottlebrush chains. In the initial stage, the bottlebrush chains are under natural extension, and then the springs are compressed before $T_{1}$ in a different degree by the applying forces, leading to the compression of the entire spring. After reaching the minimum value, it expands outward between the times $T_{1}$ and $T_{2}$, then another relaxation appears again, as shown in the Figure 2.

#### 3.1.2. Shape Factor

To describe the polymer chains comprehensively, we calculated the shape factor derived from the square radius of gyration tensor, which can be expressed as follows [43,44]:

(7) $$\langle \delta \rangle =1-3\langle \frac{L_{1}^{2}L_{2}^{2}+L_{2}^{2}L_{3}^{2}+L_{1}^{2}L_{3}^{2}}{{\left(L_{1}^{2}+L_{2}^{2}+L_{3}^{2}\right)}^{2}}\rangle$$

here, $L_{1}^{2}$, $L_{2}^{2}$, and $L_{3}^{2}$ are the three eigenvalues of the square radius of the gyration tensor. We plotted the shape factor 〈δ〉 as functions of time, as shown in Figure 3, where the dotted lines are the fitting results for convenient analysis. When 〈δ〉=1, the overall configuration tends to be rod-shaped; when 〈δ〉=0, the bottlebrush polymer is close to a spherical structure; and when 〈δ〉=0.5, a circular structure is formed [42]. The data showed that shape factors are about 0.15, indicating that the overall configuration of the bottlebrush molecule is between a spherical shape and a circular structure, so the shape of the polymer chain can be described as an ellipsoid, thus supporting the simulation results. Both shape factors underwent large oscillation and then became stable, which can be explained as the bottlebrush polymers were inserted into the phospholipid membrane when the processes began. Multiprocesses were observed in the pulling processes for both the smaller and larger pulling forces. The number of time points is the same under different tension conditions, indicating that for the shape factor, only a short time was consumed to reach a relatively stable state. However, the bottlebrush polymers underwent different fulling processes at F values of $1.7mr_{c}/\tau^{2}$ and $2.3mr_{c}/\tau^{2}$. Under a small force, the polymer shape factor underwent a similar relaxation processes as $R_{g}$, while the shape factor experienced relatively complicated processes under a large pulling force, as shown in Figure 3b. However, by comparing the time interval between $T_{1}$ and $T_{2}$ or $T_{2}$ and $T_{3}$, we will find that the shape factor in Figure 3a,b has roughly an equal time interval, which indicates that the variance of pulling force will have more effects in the gyration radius than in the shape factor. The shape factor data further interpret the spring model as the bottlebrush polymer behaves. However, the different pulling forces result in the different relaxation processes for the bottlebrush polymers, which can be supported by the results from both the shape factors and the gyration of the radius.

#### 3.1.3. Order Parameter

In the two above subsections, we studied the parameters of the bottlebrush polymers moving on the surfaces of the phospholipid membrane. In this subsection, we investigated the orientational arrangement of the phospholipid chains caused by the movement of the bottlebrush polymers. To characterize the orientational arrangement accurately, an order parameter should be introduced as follows [45,46]:

(8) $$\langle P\left(\cos\theta \right)\rangle =\langle \frac{3}{2}\cos^{2}\theta -\frac{1}{2}\rangle$$

where θ is the angle between the chain direction and the *z*-axis. An order parameter of 1 indicates that the chain is completely parallel to the *z* direction; −0.5 indicates that the chain is perpendicular to the *z* direction; and 0 represents a completely disordered distribution of the chain [32,45,46]. We showed the spatial distributions of ordering parameters for the phospholipid membrane in various pulling stages under F values of $1.7mr_{c}/\tau^{2}$ and $2.3mr_{c}/\tau^{2}$, as shown in Figure 4 and Figure 5, where the sketch maps are also listed in the right column.

Firstly, we investigated the ordering parameters under the small forces, as shown in Figure 4. In the beginning, the order parameters of the phospholipid membrane at the initial moment are approximately 0.25 over the *x* direction, as shown in Figure 4(a1), because we employed a random arrangement when generating phospholipid membrane. The side and top views in Figure 4(a2,a3) demonstrated the patterns for the bottlebrush polymers and membranes, where the polymers and membranes maintain unchanged in the initial stage of t=0τ. In the middle stage of t=40τ, the polymers were located at $x=200r_{c}$, as shown in Figure 4b. An obvious decrease in $x=200r_{c}$ was observed for the polymer ordering parameter, as shown in Figure 4(b1). Figure 4(b2,b3) showed that the bottlebrush polymers are embedded into the membranes at this position. The position of the bottlebrush molecule was caused by the adsorption of a large number of phospholipid particles, and the overall deflection angle was almost parallel to the *z*-axis, resulting in the minimum value in the position. Figure 4c shows the parameters when the bottlebrush molecule moves to the tail of the phospholipid membrane. Figure 4(c1) shows the changes of the order parameter where the bottlebrush molecule is near $x=370r_{c}$. At this stage, the phospholipid membrane has broken holes, as shown in Figure 4(c2,c3). These holes are often observed in the dynamic process of the bilayer formation, and both a stable and metastable structure can be achieved [32,47]. For the overview of the whole pulling process under $F=1.7mr_{c}/\tau^{2}$, damage occurred because of the adsorption between the polymers and membranes, and the damage positions were difficult to recover, thus causing a continuous decrease in the ordering parameter in the whole process.

Figure 5 shows the changes of the ordering parameters under $F=2.3mr_{c}/\tau^{2}$, where the sketch maps are also listed in the right column. Based on the comparison with a small pulling force, no obvious difference was observed between the two cases. Figure 5a also shows the initial states for the membranes, which were unchanged. Figure 5b shows a significant decrease in the molecular position of the bottlebrush, and a slight drop in parameter values was observed at $x=300r_{c}$. The reason is that the bottlebrush molecule has passed here, causing the partial stacking of phospholipid molecules. However, at the final stage, a difference was observed between the small and large pulling forces, as shown in Figure 5c, that is, no large parameter drop occurred at $x=0-50r_{c}$. This phenomenon occurred because the pulling force is large, causing the bottlebrush molecule to move fast. Moreover, the upper phospholipid membrane beads have been affected slightly, and the overall parameters will not substantially change. Ordered parameter measurement was used in other previous simulations and can also be used to calculate the distribution along the *z*-axis, but in our simulations, only the *x*-axis direction needs to be considered [32].

To observe the effect caused by the continues variances of pulling forces, we plotted the above parameters as a function of pulling force, as shown in Figure 6, where the pulling force varies from 0.8 to 2.4. The $R_{g}$ gradually decreased as the pulling force increased. In the spring model, this fact reflects that the spring is compressed, indicating that the overall volume of the bottlebrush molecule decreased as the force increased. However, the shape factor and the order parameter did not change substantially, and they maintained a stable value. Hence, the bottlebrush polymers maintained nearly a spherical shape with a small variance in gyration radius. The order parameters nearly remained unchanged in the whole process, indicating that the damages caused by the pulling polymers hardly affected the whole ordering arrangement.

### 3.2. Strong Adsorption

In this subsection, we discuss the interaction between the bottlebrush molecule and the phospholipid membrane in a strong adsorption case. The weak and strong adsorption cases are defined by the adsorption strength $a_{\mathrm{BM}}$, where the strong adsorptions $a_{\mathrm{BM}}=-20$ demonstrate other pulling characteristics.

#### 3.2.1. Gyration Radius

We expressed the $R_{g}$ of the bottlebrush polymers as a function of pulling times (Figure 7). Similar to the weak adsorption cases, the $R_{g}$ of the bottlebrush initially decreased, later reaching the maximum local value, and then it oscillated with a smaller amplitude, as shown in Figure 7a, where $F=1.7mr_{c}/\tau^{2}$. However, the difference was obvious for two reasons. Firstly, the oscillation amplitude under strong adsorption was larger than that under weak adsorption. In the strong case, the amplitude reached the minimum value of $\langle R_{g}\rangle =4.2r_{c}$ and then increased to a local maximum value of $\langle R_{g}\rangle =4.65r_{c}$, while in the weak case, the $\langle R_{g}\rangle$ varied within the range of $\langle R_{g}\rangle =4.1r_{c}-4.2r_{c}$. Secondly, the oscillation period was longer under strong adsorption than that under weak adsorption. Under strong adsorption, the interaction between the polymer and membrane increased. Hence, the spring needed more time to relax, thus increasing the amplitude. The bottlebrush polymer takes $T_{1}=15\tau$ to reach the minimum value and $T_{2}=45\tau$ to the local maximum value under strong adsorption. However, the polymers take approximately $T_{2}=30\tau$ under weak adsorption. Under the large pulling force of $F=2.3mr_{c}/\tau^{2}$ (as shown in Figure 7b), although the polymers exhibited a similar oscillation behavior to the small pulling forces, the polymer size underwent a richer process than that under weak adsorption, where the polymer exhibited a nearly monotonous decrease in $\langle R_{g}\rangle$. By comparing this with other works, the change of $\langle R_{g}\rangle$ of a single bottlebrush molecule remained different [34]. Another important feature is that the strong adsorption between the polymer and membrane increased the time to travel across the whole membrane because of the slower movement for the polymers, and the broken hole will appear in the membrane when the polymers are traveling for enough time, where $T_{e}=120\tau$ and $T_{e}=90\tau$ under strong adsorption with forces of $F=1.7mr_{c}/\tau^{2}$ and $F=2.3mr_{c}/\tau^{2}$, while $T_{e}=75\tau$ and $T_{e}=55\tau$ under weak adsorption with forces of $F=1.7mr_{c}/\tau^{2}$ and $F=2.3mr_{c}/\tau^{2}$. This behavior can also be explained by the spring model, but the strong adsorption interaction leads to more obvious effects in the relaxation processes.

#### 3.2.2. Shape Factor

The variance of the shape factors for the bottlebrush polymers in the strong adsorption state are shown in Figure 8. For a small pulling force of $F=1.7mr_{c}/\tau^{2}$, as shown in Figure 8a, the shape factors oscillated with a small and obvious period, indicating that the shape factor is sensitive under the pulling process. This phenomenon is caused by the definition of the shape factor, which considers the higher dimension. The large force of $F=2.3mr_{c}/\tau^{2}$ (Figure 8b) shows oscillation, similar to the case of the small forces. However, the oscillation behavior is more obvious with a larger amplitude. In comparison with the weak adsorption cases, the shape factors exhibit a similar behavior. The shape factors initially increased and reached the local maximum value at $T_{1}$ and decreased to the local minimum value at $T_{2}$. The relaxation sustained a longer time in the strong adsorption cases compared with the weak adsorption case. The variance in the shape factor of a single bottlebrush molecule is different from those in previous works [42], in which the shape factor had undergone a process of first decreasing and then increasing, which is contrary to our results.

#### 3.2.3. Order Parameter

The order parameters of the phospholipid membrane under strong adsorption were discussed, as shown in Figure 9 and Figure 10, at $F=1.7mr_{c}/\tau^{2}$ and $F=2.3mr_{c}/\tau^{2}$, respectively, where three different time points were selected to demonstrate the entire movement process. At the initial stage, as shown in Figure 9a, the distributions of order parameters under the small force of $F=1.7mr_{c}/\tau^{2}$ were similar to those under weak adsorption under the same pulling forces. Figure 9(b1) shows that the ordering parameter had the minimum value at the position of the bottlebrush polymer. Hence, the bottlebrush polymer damaged the arrangement of membrane chains as it passed this position. The damaged area under strong adsorption is smaller than that under weak adsorption, as shown in Figure 4b, due to the strong adsorption between the polymer and membrane. Figure 9(c1) shows that the phospholipid membrane has a broken hole, and this result differs from that under weak adsorption, as shown in Figure 4c. A large fluctuation was observed in the order parameters because of the movement of the bottlebrush polymer, but no obvious change was observed in the effect of bead stacking at the broken position.

Figure 10 shows the distribution of the order parameters for the membrane with the large force $F=2.3mr_{c}/\tau^{2}$. Similarly, the initial stages are shown in Figure 10a, while the other typical stages are shown in Figure 10b,c. The ordering parameters have similar distributions at these two stages, and no obvious change was observed in the membrane breakage. Based on the analysis of the above data, whether the phospholipid membrane is broken or not will not affect the order parameter. Therefore, the size of the pore has no obvious regularity and shows randomness, which is different from the results in the previous works, where the pore formations are under tension [48,49,50,51]. The obvious difference is that the ordering parameter is distributed randomly at the membranes because of the movement of bottlebrush polymers.

To further understand the effect of pulling forces on the interaction systems, we investigated the continuous variance of pulling forces for the polymers and plotted the system parameters as functions of pulling forces, as shown in Figure 11. The pulling force varied from $0.8mr_{c}/\tau^{2}$ to $2.4mr_{c}/\tau^{2}$, similar to the weak adsorption cases. The $R_{g}$ increased slowly with increasing pulling force, and this result differs from the weak adsorption cases, where $R_{g}$ gradually decreased. However, $R_{g}$ gradually increased under strong adsorption. This can be understood as a spring modelled by the bottlebrush polymers. Under weak adsorption, the initial state is relaxed, and the spring was compressed when the pulling force increased. This compression led to the decrease in volume, where $R_{g}$ decreased. However, for the strong adsorption case, the initial bottlebrush polymers can be regarded as the beginning in a state of extreme compression under an external force. The spring naturally bounces outwards, thus increasing the volume and $R_{g}$. However, the order parameter and shape factor did not change with the change of pulling force, as shown in Figure 11. The average shape factor is approximately 0.2 under strong adsorption, and this value is higher than those under weak adsorption. Hence, the polymers are more asymmetric under strong adsorption.

## 4. Conclusions

We carried out a DPD simulation on the interaction between the bottlebrush polymer and supported phospholipid membrane in solutions based on a CG model. We considered the weak and strong adsorption between the bottlebrush polymers and phospholipid membranes. The system parameters, such as the $R_{g}$ and shape factors for the polymers, and the order parameters for the membranes were investigated and compared.

Under weak and strong adsorption, the $R_{g}$ of the bottlebrush polymers initially decreased and then stabilized after a period of oscillation. A spring model can be introduced to explain this phenomenon, where the bottlebrush polymers act like an expanding and contracting spring as it is dragged. Under weak adsorption, the whole spring gradually shrinks, whereas under strong adsorption, the whole spring gradually expands because the initial state of the spring differs under different adsorption cases. We also investigated the changes in the shape factors of the bottlebrush polymers during the pulling processes. The results showed that the spring had a nearly spherical shape, which is not affected by the strength of adsorption. The order parameters of the phospholipid membrane indicate that the change only appeared in the position of the bottlebrush polymers; no obvious change was observed in the membrane puncture, and it was not affected by the adsorption strength.

We expect a richer pulling behavior when the parameters of the system are set beyond the values used in this study. For example, we assumed other parameters for the bottlebrush polymer in this work. In the case of the different bottlebrush polymer with a different mainchain length, $N_{M}$, different number of sidechains, $n_{S}$, and different sidechain length, $N_{S}$, we expect that the pulling process can be distinctive. In the present work, we considered all the sidechains as the same types. The pulling processes for other types of bottlebrush polymers [52], or even the bottlebrush copolymers that the experiments investigated [53], which were on the surfaces of the membranes, would be an interesting research focus. However, it is expected that the other manners of pulling forces, such as the pulling forces only applying on the side chains, will lead to the strong effects on the polymer shapes. Our observation on the subset of full parameter space is a starting point to explore the pulling behavior of polymer system that interacts with the phospholipid membranes in the multidimensional parameter space.
